# Optimising metameric spectra for integrative lighting to modulate the circadian system without affecting visual appearance

**DOI:** 10.1038/s41598-021-02136-y

**Published:** 2021-11-30

**Authors:** Babak Zandi, Oliver Stefani, Alexander Herzog, Luc J. M. Schlangen, Quang Vinh Trinh, Tran Quoc Khanh

**Affiliations:** 1 Department of Electrical Engineering and Information Technology, Laboratory of Lighting Technology, Technical University of Darmstadt, Darmstadt, Germany; 2 Transfaculty Research Platform Molecular and Cognitive Neurosciences (MCN), Centre for Chronobiology, University of Basel, Basel, Switzerland; 3 Department Human-Technology, Intelligent Lighting Institute, Eindhoven University of Technology, Eindhoven, The Netherlands

**Keywords:** Visual system, Circadian rhythms and sleep, Lasers, LEDs and light sources

## Abstract

Smart integrative lighting systems aim to support human health and wellbeing by capitalising on the light-induced effects on circadian rhythms, sleep, and cognitive functions, while optimising the light’s visual aspects like colour fidelity, visual comfort, visual preference, and visibility. Metameric spectral tuning could be an instrument to solve potential conflicts between the visual preferences of users with respect to illuminance and chromaticity and the circadian consequences of the light exposure, as metamers can selectively modulate melanopsin-based photoreception without affecting visual properties such as chromaticity or illuminance. This work uses a 6-, 8- and 11-channel LED luminaire with fixed illuminance of 250 lx to systematically investigate the metameric tuning range in melanopic equivalent daylight illuminance (EDI) and melanopic daylight efficacy ratio (melanopic DER) for 561 chromaticity coordinates as optimisation targets (2700 K to 7443 K ± Duv 0 to 0.048), while applying colour fidelity index R_f_ criteria from the TM-30-20 Annex E recommendations (i.e. R_f_ $\ge$ 85, R_f,h1_ $\ge$ 85). Our results reveal that the melanopic tuning range increases with rising CCT to a maximum tuning range in melanopic DER of 0.24 (CCT: 6702 K, Duv: 0.003), 0.29 (CCT: 7443 K, Duv: 0) and 0.30 (CCT: 6702, Duv: 0.006), depending on the luminaire’s channel number of 6, 8 or 11, respectively. This allows to vary the melanopic EDI from 212.5–227.5 lx up to 275–300 lx without changes in the photopic illuminance (250 lx) or chromaticity ($\Delta u'v'$ $\le$ 0.0014). The highest metameric melanopic Michelson contrast for the 6-, 8- and 11-channel luminaire is 0.16, 0.18 and 0.18, which is accomplished at a CCT of 3017 K (Duv: − 0.018), 3456 K (Duv: 0.009) and 3456 K (Duv: 0.009), respectively. By optimising ~ 490,000 multi-channel LED spectra, we identified chromaticity regions in the CIExy colour space that are of particular interest to control the melanopic efficacy with metameric spectral tuning.

## Introduction

Light interventions with an increased spectral irradiance within the short-wavelength range can enhance melatonin suppression, task-related performance, heart rate, body temperature and alertness^1–4^. Under photopic lighting conditions, such responses are mostly driven by the short-wavelength spectral range of a light stimulus, triggering the M1- and M2-subtypes of the melanopsin containing, intrinsically photosensitive retinal ganglion cells (ipRGCs) in the outer retina^5,6^. The ipRGCs drive the pupillary light reflex^7–10^ and circadian responses to light through their projections to the olivary pretectal nucleus and suprachiasmatic nuclei in the brain^5,11–13^. Since the discovery of the ipRGCs, one practical challenge in workplace lighting is to design lighting conditions that are perceived as white and can secure an appropriate balance of requirements with respect to visual aspects while simultaneously enabling for a selective spectral modulation of the light’s effectiveness to drive circadian photoentrainment^14–16^. The LED technology allows to engineer and tune white polychromatic spectra, by coating blue-emitting diodes with a phosphor layer^17,18^ or by using multi-channel luminaires in which the wavelength distributions of different LED types are combined and additively mixed.

In modern interior lighting, multi-channel LED luminaires are of interest as they can dynamically adapt the spectral power distribution, and hence adjust colour rendering, chromaticity coordinates or chroma of an illuminated object. This kind of technology is an essential tool in smart lighting systems, allowing occupants to manually adjust the correlated colour temperature (CCT) or other (visual) characteristics depending on the observers' preferences or task^19^. However, at particular times of day (for instance, during the 2–3 h before bedtime or during sleep), chromaticity choices at higher CCTs, which might also increase the light exposure in the short-wavelength range, are not recommended because of their enhanced effectivity to suppress melatonin and increase alertness^20–22^. Thus, there could be a conflict between the users’ visual preferences of chromaticity and the degree of triggering circadian responses if other crucial metrics such as the time of light exposure and light intensity remain steady.

Furthermore, the next generation of intelligent smart lighting systems will adapt the light settings such as CCT and illuminance automatically with data-driven methods^23–28^ to match the user’s individual lighting preference, presumably without addressing recommendations for circadian lighting^19^. As a solution, metamer spectra could be used, capable of keeping visual appearance (chromaticity, CCT or (il)luminance) steady while tuning non-image-forming metrics silently in the background for actively influencing the humans' physiological response^19,29^. For example, a set of multi-channel LED spectra can be optimised for a given chromaticity coordinate at a fixed (il)luminance using heuristic or gradient-based optimisation methods^30–32^. Then, the two pairs of spectra with the maximum difference in ipRGCs excitation can be selected. When applying the two metamer spectra to an illumination system, the circadian system can be modulated without altering the visual sensation^33^. For general lighting applications and continuous, sustained light exposure, the magnitude of a circadian response can be accurately predicted based on the melanopic stimulation within a particular (metameric) spectrum^34^.

A recent work by Souman et al. showed that metameric spectra that differentially target melanopsin photoreception at a fixed CCT and illuminance (in this case 2700 K and 175 lx) can reduce human melatonin production by 50%, but did not result in differences in the reported alertness^35^. The colour rendering index (CRI) R_a_ of the used stimuli, however, was lower than 80, meaning that it was not suitable for interior lighting. The two metameric spectra used by Souman et al. differed in their melanopic equivalent daylight illuminance (melanopic EDI) ${E}{}_{mel}^{D65}$ between a ${E}{}_{mel,Min}^{D65}$ 54.6 lx and a ${E}{}_{mel,Max}^{D65}$ of 188.8 lx^34,36^. Hence, the difference in melanopic equivalent daylight illuminance between the applied metameric pair (${\Delta E}{}_{mel}^{D65}$) was 122 lx. Another study by Allen et al. investigated the impact of metamerism on the circadian system with a custom-built 5-channel display unit^33^. It was found that the melatonin level and the reported sleepiness can be varied without altering the visual appearance of light by differentially modulating ipRGC stimulation between two metameric stimuli with a ${E}{}_{mel,Min}^{D65}$ of 24.7 lx and a ${E}{}_{mel,Max}^{D65}$ of 77.7 lx, resulting in a melanopic modulation ${\Delta E}{}_{mel}^{D65}$ of 52.9 lx. Further, an ongoing study investigates light effects of metameric visual display that differs in melanopic irradiance (ca threefold) but matches in cone excitation. Preliminary analysis yields an overall alerting response in the high melanopic versus the low melanopic condition, concomitant with a trend of reduced melatonin levels in the high melanopic condition^37^.

Before metamer spectra can be used, spectral optimisation must be performed, which determines the dimming levels of each LED channel in a luminaire. The higher the number of LED channels, the more likely it is that metamer solutions can be found for a single chromaticity coordinate at a given (il)luminance. Thus, the key of metamerism in circadian photoentrainment is the art of spectral optimisation, which aims to design suitable spectra with an appropriate change in melanopic-content. Spectral optimisation procedures are time-consuming and computationally intensive. Therefore, it is necessary to know which chromaticity coordinates yield the largest melanopic metameric tuning range when optimising spectra for a selected CCT. Further, the knowledge of chromaticity regions with higher melanopic metameric tuning range could support the choice of a spectral optimisation method. For instance, if a gradient-based optimisation approach is applied, metameric spectra could not be considered in the solutions, carrying the risk of randomly optimising a spectrum with a sub-optimal melanopic efficacy at a given chromaticity.

A recent spectral optimisation simulation study by Zandi et al. revealed that the melanopic metameric tuning ranges are not uniformly distributed around the Planckian locus^19^. It was reported that the highest relative melanopic tuning range can be reached near the Planckian locus between 3292 and 4717 K within a Duv range of -0.009 and 0.006. In such a CCT range, the melanopic stimulus at a particular chromaticity coordinate and fixed luminance can be modulated by up to 65%, using metameric spectra ($\Delta u'v'\le 7.05 \cdot {10}^{-5}, \Delta L \le 1\mathrm{cd}/\mathrm{m}^2$). Furthermore, Aderneuer et al.^38^ used a 6-channel LED luminaire to design metameric light with a CCT of 3700 K and found that the non-SI compliant metric circadian stimulus (CS) could be varied between 0.20 and 0.41.

It is currently unknown to what extent a melanopic-stimulus can be modulated through metameric spectra and how the difference in melanopic equivalent daylight illuminance ${\Delta E}{}_{mel}^{D65}$ of metameric pairs are distributed in the colour space when the photopic illuminance is fixed. Such an information is relevant for developing lighting systems in research and industrial applications, as there are efforts to achieve a higher melanopic efficacy while maintaining the CCT^30,39^. This work optimised 490,068 spectra for 561 chromaticity coordinate targets along the Planckian locus, which is one of the largest available spectral metamer datasets, at the authors’ best knowledge. We report the first systematic investigation to identify metameric pairs with the largest melanopic tuning range for various choices of chromaticity coordinates across the colour space. For this, metamer spectra were used with a fixed photopic illuminance E_v_ (250 lx), pre-defined chromaticity coordinates and a IES color fidelity index^40^ R_f_ above either 85 or 90. The melanopic metameric tuning ranges were mapped in the CIExy colour space, allowing for an intuitive way to assess the chromaticity ranges where an appropriate spectral design can produce large melanopic modulations via metameric stimuli.

## Results

**Figure 1:**
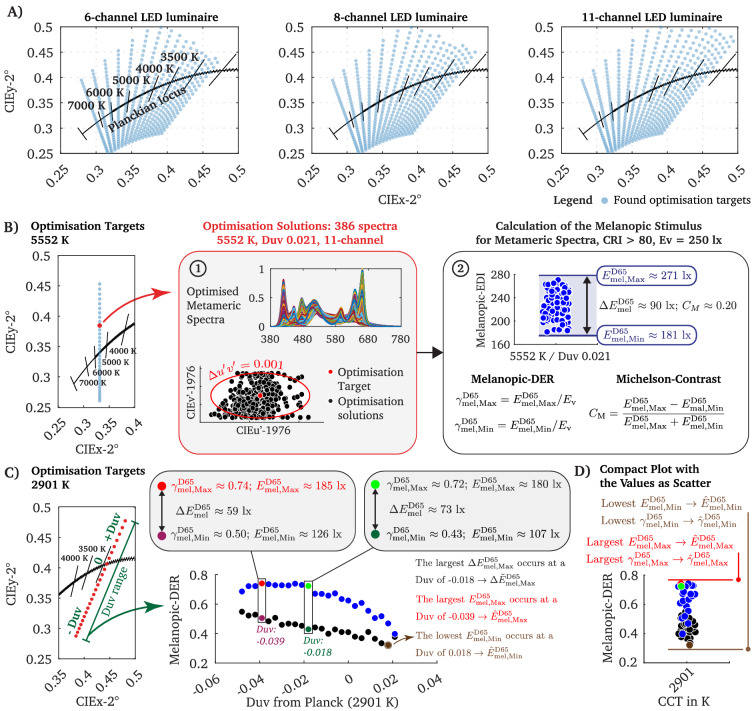
The chromaticity coordinates for which spectra where successfully optimised and strategies of assessing the non-visual quality of the metameric spectra. (**A**) We used a uniform grid of 561 chromaticity coordinates along the Planckian locus (2700 K to 7443 K ± Duv 0 to 0.048 with Duv-steps of 0.003) as optimisation targets for spectral optimisation. Depending on the LED luminaire’s channel configuration, the number of successfully optimised spectra per chromaticity target differs. The 6-channel LED luminaire, for example, was unable to yield spectra for chromaticity targets with higher Duv values at CCTs between 2700 and 3000 K. The blue scatter points in the CIExy-2° colour space highlight the chromaticity coordinates for which metameric spectra were successfully optimised. (**B**) For each of the blue points in the CIExy-2° colour space (left panel), numerous metameric spectra were available. When using a single chromaticity coordinate at 5552 K (Duv of 0.021, red point in the left panel) as an optimisation target for the 11-channel luminaire, 386 spectra were available (see panel 1). We defined spectra whose chromaticity coordinates were inside the tolerance range of $\Delta u',\Delta v'\le 0.001$ (to the optimisation target) as metameric spectra (see panel 1). From the remaining spectra the upper and lower limit of ${E}{}_{mel}^{D65}$ can be classified, representing the melanopic tuning range $\Delta {E}{}_{mel }^{D65}$ for the single chromaticity coordinate (see panel 2). Each chromaticity coordinate target is characterised with the respective ${E}{}_{mel, Min}^{D65}$, ${E}{}_{mel, Max}^{D65}$, $\Delta {E}{}_{mel}^{D65}$, ${\gamma }{}_{mel,Min}^{D65}$, ${\gamma }{}_{mel,Max}^{D65}$, $\Delta {\gamma }{}_{mel}^{D65}$ and its melanopic Michelson Contrast $C_{M}$. (**C**) When considering the chromaticity coordinates for one CCT (left panel), the upper (blue points) and lower limit (black points) of the melanopic DER (${\gamma }{}_{mel,Min}^{D65}$, ${\gamma }{}_{mel,Max}^{D65}$) can be plotted and analysed as a function of the Duv steps, making possible to state which Duv distance to Planck is ideal for spectral optimisation. The largest or lowest melanopic EDI (and respective melanopic DER) values of a CCT (across all Duv values) at a photopic illuminance of 250 lx will be denoted as ${\widehat{E}}{}_{mel, Min}^{D65}$ (brown point), $\widehat{E}{}_{mel, Max}^{D65}$ (red point). As the melanopic EDI values correlate with the melanopic DER, these key points can also be expressed with $\widehat{\gamma}{}_{mel,Min}^{D65}$ and $\widehat{\gamma }{}_{mel,Max}^{D65}$. The largest melanopic tuning range of a CCT will be denoted as $\Delta \widetilde{E}{}_{mel}^{D65}$ or for a generalised description as ${\Delta \widetilde{\gamma}}{}_{mel}^{D65}$. (**D**) To compare the non-visual metrics across the different CCT steps, we contracted the values for each Duv value to one axis with blue points as the upper limit of the melanopic DER tuning range and black points for the lower limit. The red point states the $\widehat{\gamma}{}_{mel,Max}^{D65}$ and accordingly the $\widehat{E}{}_{mel, Max}^{D65}$ value (see panel **C)**).

We used a predefined uniform grid of 561 chromaticity coordinates (CIEu’v’-1976, Supplementary Materials Fig. S1) along the Planckian locus (2700 K to 7443 K ± Duv 0 to 0.048 with Duv-steps of 0.003)^19,41^. For each chromaticity target, spectra were optimised using a heuristic optimisation procedure for a 6-channel, 8-channel, and 11-channel LED luminaire. Spectra within the tolerance range of $\Delta u',\Delta v'\le 0.001$ to the objective were classified as metamers. With the 6-channel LED configuration, spectra were found for 464 of 561 chromaticity targets (Fig. 1A). For each chromaticity in average, 281 $\pm$ SD 30.18 metamers were optimised, leading to a total of 130,385 spectra. With the 8-channel LED configuration, we optimised a total of 172,693 spectra with an average of 364 $\pm$ SD 56.85 metamers for 474 chromaticity targets (Fig. 1A). The optimisation of the 11-channel LED luminaire reached a total of 186,990 spectra with a mean of 371 $\pm$ SD 61.57 metamers for each of the 504 found chromaticity targets (Fig. 1A). In sum, 490,068 spectra were optimised, which is the second-largest published metamer spectral dataset^19^ for multi-channel LED luminaires, at the authors’ best knowledge. For the analysis, the optimised spectra were linearly scaled to a fixed photopic illuminance E_v_ of 250 lx.

### Approaches of evaluating the melanopic effectiveness of metameric spectra

The principle of univariance states that single cones are colour blind^42,43^, meaning visual perception results from the antagonistic cone-opponency between the outer photoreceptors. This physiological phenomenon is the basis of metameric spectra, as different spectral power distributions can trigger the same visual response if the excitations across the photoreceptors do not change among the stimuli^43^. In colourimetry, however, metameric spectra are defined as those whose chromaticity coordinates and luminance (or illuminance) match within a specified tolerance range. As the humans’ non-visual system works approximately independent of the visual pathway, it is possible to leverage such stimuli and modulate circadian responses without significantly affecting the chromaticity or luminous sensation. For example, at a CCT of 5552 K (Duv: 0.021, 11-channel), we optimised 386 spectra, whose chromaticity coordinates are located within the tolerance range of $\Delta {u'},\Delta v'\le 0.001$ (Fig. 1B, point 1). Subsequently, we used the melanopic EDI^34,36^ ${E}{}_{mel}^{D65}$ to quantify the impact of the optimised metameric light spectra on the non-visual pathway, e.g. photic regulation of physiological and circadian responses in humans^44^. This metric describes the melanopsin activated ipRGC response^1^ without the extrinsic synaptic input from outer retinal photoreceptors to the ipRGCs^14,45^. According to common interior lighting recommendations^21^, we considered those spectra that fulfil a specified colour rendering metric, which is in Fig. 1B (point 2), for example, a CRI R_a_ higher than 80. From the set of optimised spectra, the upper and lower limit of the melanopic EDI with ${E}{}_{mel, Max}^{D65}$ and ${E}{}_{mel, Min}^{D65}$ can be extracted (Fig. 1B, point 2). From the remaining two metameric pairs, we calculated the maximum possible change of the melanopic EDI for this specific chromaticity point with $\Delta {E}_{mel}^{D65}= |{E}_{mel, Max}^{D65}- {E}_{mel, Min}^{D65}|$. As shown in Fig. 1B point 2, using a CCT of 5552 K and Duv of 0.021, the melanopic EDI can be varied from 181 to 271 lx ($\Delta {E}{}_{mel}^{D65}\approx$ 90 lx), corresponding to a melanopic Michelson (see “Methods”) contrast $C_{M}$ of ~ 0.20, although the photopic illuminance remains steady at 250 lx. Previous works on optimising highly effective non-visual spectra^30,39,46^ aimed to catch the ${E}{}_{mel, Max}^{D65}$ stimuli or other metrics that correlate with the upper limit, while in metameric optimisation, the lower limit must also be evaluated to assess the maximum tuning range $\Delta {E}{}_{mel}^{D65}$ and its corresponding melanopic Michelson contrast ${C}{}_{M}$ for a particular chromaticity and photopic illuminance.

Apart from stating the tuning range of the melanopic illuminance, the ratio between ${E}{}_{mel}^{D65}$ and $E_{v}$ is a standard metric for assessing the melanopic efficacy of spectra, denoted as the melanopic daylight efficacy ratio ${\gamma }{}_{mel}^{D65}$ (melanopic DER)^14,36^. The value ${\gamma }{}_{mel}^{D65}$ can be used as a factor to calculate ${E}{}_{mel}^{D65}$ as a function of any photopic illuminance. If, for example, ${\gamma }{}_{mel}^{D65}$ is 0.8 for a given spectrum, then a photopic illuminance of 500 lx could achieve a ${E}{}_{mel}^{D65}$ of 400 lx, calculated with the formula ${E}{}_{mel}^{D65}={E}_{v}\cdot {\gamma }{}_{mel}^{D65}$. By knowing a melanopic DER value, the photopic illuminance that is required to get a specific ${E}{}_{mel}^{D65}$ can be calculated. This makes ${\gamma }{}_{mel}^{D65}$ a highly useful quantity for scientific analyses, as the metric provides a generalised description uncoupled from a pre-specified photopic illuminance level. Therefore, instead of expressing the melanopic tuning of a particular chromaticity point in terms of $\Delta {E}{}_{mel}^{D65}$, it is a good scientific practise to express the tuning range in terms of its $\Delta {\gamma }{}_{mel}^{D65}$ value, which can be calculated using the upper and lower limit of the melanopic DER according to $|{\gamma }_{mel, Max}^{D65}- {\gamma }_{mel, Min}^{D65}|$ (see Fig. 1B, point 2). The tuning range in melanopic illuminance $\Delta {E}{}_{mel}^{D65}$ for a particular photopic illuminance ${E}{}_{v}$ can be calculated using the formula ${\Delta E}{}_{mel}^{D65}={E}_{v}\cdot {\Delta \gamma }{}_{mel}^{D65}$. Note that a melanopic DER ${\gamma }{}_{mel}^{D65}$ greater than one indicates that the melanopic equivalent daylight (D65) illuminance is higher than the (photopic) illuminance of the source. For the standard illuminant D65, the melanopic DER equals to one. It should be mentioned, that knowing ${\Delta E}{}_{mel}^{D65}$ of metameric pairs is not sufficient to predict circadian responses. For instance, melatonin suppression is a function of ${E}{}_{mel}^{D65}$ and follows a sigmoidal function that saturates at high melanopic equivalent daylight illuminances^34^. Thus, in addition to the melanopic tuning with $\Delta {E}_{mel}^{D65}= |{E}_{mel, Max}^{D65}- {E}_{mel, Min}^{D65}|$, the starting point ${E}{}_{mel, Min}^{D65}$ is of interest for evaluating the circadian effectiveness, which indicates to what extend the melatonin suppression can be modulated without altering visual metrics.

In this work, however, the spectral dataset of metamer spectra was optimised for each chromaticity target from which a spectrum with ${E}{}_{mel, Min}^{D65}$ and one with ${E}{}_{mel, Max}^{D65}$ can be identified. Therefore, the melanopic DER can be calculated for the upper and lower limit of ${E}{}_{mel}^{D65}$ with ${\gamma }{}_{mel,Max}^{D65}$ and ${\gamma }{}_{mel,Min}^{D65}$ for each chromaticity target (Fig. 1B, point 2). Additionally, the melanopic EDI of the two metameric pairs can be used to calculate the melanopic Michelson contrast $C_{M}$ (see “Methods”). As numerous spectra were calculated for each chromaticity target, we can even evaluate the spectral optimisation limit for non-visual purposes, making it possible to state for which Duv value of a CCT the highest ${\Delta E}{}_{mel}^{D65}$ (or generalised ${\Delta \gamma }{}_{mel}^{D65})$ can be achieved (Fig. 1C).

For each CCT, numerous chromaticity coordinate targets were used, meaning different Duv distances to the Planckian locus (Fig. 1C). One essential question in optimisation procedures is, which chromaticity coordinate should be chosen along the Duv distances to reach a spectrum with the largest melanopic metameric tuning range. As we determined the ${E}{}_{mel, Min}^{D65}$ and ${E}{}_{mel, Max}^{D65}$ values for different chromaticity points, the upper and lower limits of the melanopic DER (${\gamma }{}_{mel,Max}^{D65}$, ${\gamma }{}_{mel,Min}^{D65}$) is known across the Duv steps for each CCT. In Fig. 1C, it can be observed that with a Duv of − 0.018 (2901 K), the largest melanopic DER change can be achieved, resulting in a ${\Delta \gamma }{}_{mel}^{D65}$ of 0.29. For example, when using a photopic illuminance of 250 lx, this would correspond to a metameric melanopic EDI change ${\Delta E}{}_{mel}^{D65}$ of ~ 73 lx (see Fig. 1C, middle panel). However, if the objective is to optimise spectra with the largest non-metameric melanopic efficacy $\widehat{\gamma}{}_{mel,Max}^{D65}$ at a particular fixed CCT, a chromaticity coordinate with a Duv of -0.039 should be preferred as an optimisation target, leading to a ${\gamma }{}_{mel, Max}^{D65}$ of 0.74, but whose metameric tuning ${\Delta \gamma }{}_{mel}^{D65}$ value is lower with 0.236 (${\Delta E}{}_{mel}^{D65}\approx 59$ lx). Thus, when selecting the ideal chromaticity coordinate (fixed CCT) for spectral optimisation, it must be decided whether the objective is to maximise or minimise the melanopic DER ${\gamma }{}_{mel}^{D65}$ or to find a metameric pair with the largest melanopic tuning range ${\Delta \gamma }{}_{mel}^{D65}$. In the following, the largest melanopic DER of a particular CCT will be denoted as $\widehat{\gamma}{}_{mel, Max}^{D65}$, its lowest melanopic DER as $\widehat{\gamma}{}_{mel, Min}^{D65}$ and its largest melanopic tuning range across all Duv values as ${\Delta \widetilde{\gamma }}{}_{mel}^{D65}$ (see Fig. 1C and D).

As discussed, the objective of this work is to analyse the melanopic tuning range of metameric spectra while ensuring a good colour rendition. The colour rendition of a light source is an essential aspect of integrative lighting and can influence the subjectively evaluated naturalness, vividness and preference of an object’s colour in an illuminated environment^47^. In this work, we evaluated the colour rendition of the optimised light spectra by considering only the aspect of colour fidelity, using the ANSI/IES colour fidelity index R_f_ (TM-30-20) as metric^40^. The colour fidelity index R_f_ is in its characterisation intent analogous to the general colour rendering index^47^ (CRI) R_a_. However, the latter (CRI R_a_) has several shortcomings when evaluating artificial polychromatic light that is mixed with narrowband LED spectra (see the works^47–52^ for further explanation), which is why the colour fidelity index R_f_ is recommended in the CIE 224:2017^53^ for scientific use cases. To select appropriate R_f_ threshold conditions, we have followed the TM-30-20 Annex E recommendations^40^, which specify distinct criteria for the categories colour preference, colour vividness, and colour fidelity, separated into three priority levels, respectively^40,47^. These criteria can be applied for polychromatic LED spectra in illuminated environments with a photopic illuminance between 200 and 700 lx^40,47^. The intended design goal in this work is to ensure a good colour fidelity of the optimised light spectra. For this, we have chosen the priority level three (R_f_ $\ge$ 85, R_f,h1_ $\ge$ 85) and level two (R_f_ $\ge$ 90, R_f,h1_ $\ge$ 90) from the fidelity category, where R_f,h1_ is defined as the local colour fidelity of the first bin in the colour vector graphic^40^. In the next section, the analysis of the optimised spectra will be carried out with the discussed strategy and metrics. To state the melanopic DER range for a particular CCT, we plotted all computed melanopic DER values for the various Duv steps of a single CCT as a single category (Fig. 1D).

### The melanopic DER tuning range of metameric spectra increases with higher CCTs

**Figure 2:**
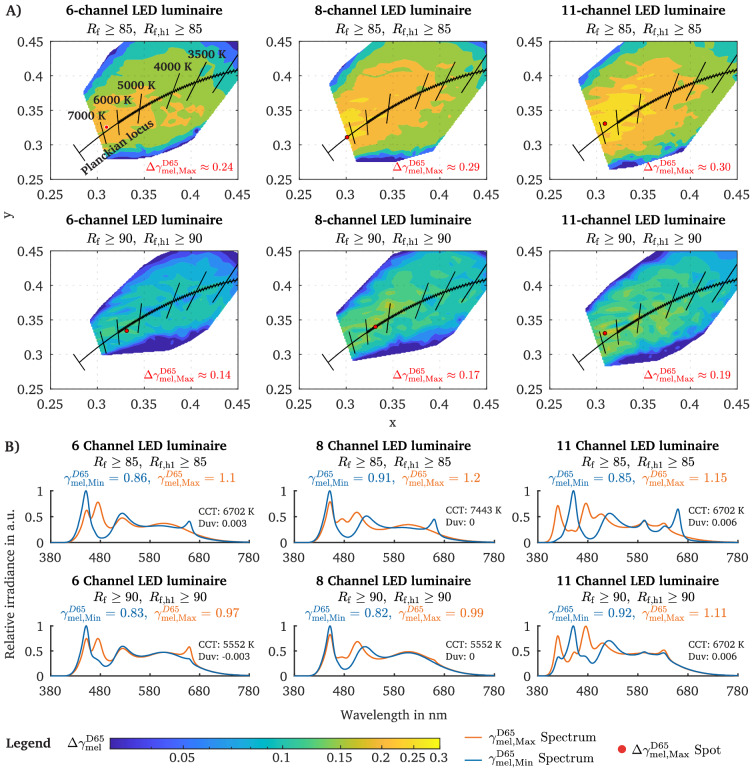
Melanopic metameric tuning range $\Delta {\gamma }{}_{mel }^{D65}$ across various CIExy coordinates. (**A**) The melanopic tuning range $\Delta {\gamma }{}_{mel }^{D65}$ in the CIExy-2° colour space for three LED luminaire settings (6, 8 and 11 primaries) and two different IES colour fidelity R_f_ conditions with (*R*_*f*_ $\ge$ 85*, R*_*f,h1*_ $\ge$ 85*)* and (*R*_*f*_ $\ge$ 90*, R*_*f,h1*_ $\ge$ 90*)*. The red dots signal the CIExy coordinates at which the melanopic tuning range is maximal across all CCT and Duv steps ($\Delta {\gamma }{}_{mel, Max}^{D65}$). The respective value of the largest melanopic tuning range $\Delta {\gamma }{}_{mel, Max}^{D65}$ is reported in the lower left of each metameric map plot. (**B**) The maximum tuning range $\Delta {\gamma }{}_{mel, Max}^{D65}$ (red dot, panel **A**) is derived from subtracting the ${\gamma }{}_{mel, Max}^{D65}$ and ${\gamma }{}_{mel, Min}^{D65}$ values of the spectral pair that corresponds to the red dot. The two spectra of the metameric pair, which maximise the melanopic tuning range ($\Delta {\gamma }{}_{mel, Max}^{D65}$), are plotted for each luminaire setting and applied colour fidelity conditions.

For each of the investigated chromaticity targets (see Fig. 1A), we calculated the maximum melanopic tuning range, which we defined as $\Delta {\gamma }_{mel}^{D65}= |{\gamma }_{mel, Max}^{D65}- {\gamma }_{mel, Min}^{D65}|$. Accordingly, the melanopic tuning range $\Delta {\gamma }{}_{mel}^{D65}$ was mapped in the CIExy colour space for each defined fidelity criterium (Fig. 2A). The results indicate that, regardless of the LED channel number, chromaticity coordinate and fidelity criterium, the largest melanopic tuning range $\Delta {\gamma }{}_{mel,Max}^{D65}$ across all CCTs varies between 0.14 to 0.30 (Fig. 2A). This tuning range can be achieved for the different luminaire configurations without altering visual metrics such as chromaticity or photopic illuminance. Furthermore, the largest melanopic tuning range across all CCTs can be reached for chromaticity coordinates that are above Planck (Duv 0 to 0.006) and correspond to the CCTs 6702 K (6-channel, Duv: 0.003), 7443 K (8-channel, Duv: 0) and 6702 K (11-channel, Duv: 0.006) if a colour fidelity condition of R_f_ $\ge$ 85 and R_f,h1_ $\ge$ 85 (priority level three) is applied (see red dots in Fig. 2A).

However, with rising CCT, the melanopic tuning range $\Delta {\gamma }{}_{mel}^{D65}$ increases, while a saturation effect is notable from approximately ~ 4500 K onwards, meaning that at a specific CCT threshold that additionally depend on the LED channel number, $\Delta {\gamma }{}_{mel}^{D65}$ only marginally increases (see Supplementary materials, Fig. S2). Note that the melanopic tuning range at steady CCT differs between the various Duv values (see Fig. 2A). When using an 8-channel or 11-channel LED luminaire (R_f_ $\ge$ 85, R_f,h1_ $\ge$ 85), the (Duv) area in which a melanopic tuning range $\Delta {\gamma }{}_{mel}^{D65}$ of at least 0.2 ($\Delta {E}{}_{mel}^{D65}$ = 50 lx for $E_{V}$= 250 lx) can be reached increases (Fig. 2A). If the colour fidelity criterion is set to the priority level two (R_f_ $\ge$ 90, R_f,h1_ $\ge$ 90), then the largest melanopic tuning range ${\Delta {\gamma }}{}_{mel, Max}^{D65}$ across all CCT steps remains between 0.14 and 0.19, depending on the used LED configurations (Fig. 2A). However, from about 3000 K upwards, the ${\Delta {\gamma }}{}_{mel}^{D65}$ saturates and becomes approximately independent of the CCT when using the second priority level (R_f_ $\ge$ 90, R_f,h1_ $\ge$ 90) of the fidelity criterion (Supplementary materials, Fig. S2). In Fig. 2B, the relative metamer spectra pairs that could cause the largest melanopic tuning range ${\Delta {\gamma }}{}_{mel, Max}^{D65}$ are shown (see red dots in Fig. 2A). It merits to be noted that the melanopic DER (and hence the melanopic EDI) can be tuned by increasing the blue LED-channel’s duty cycle ($\lambda_{Peak}= 470$ nm) and adapting the other (opposite) channels to maintain the chromaticity point and photopic illuminance (Fig. 2B).

**Figure 3:**
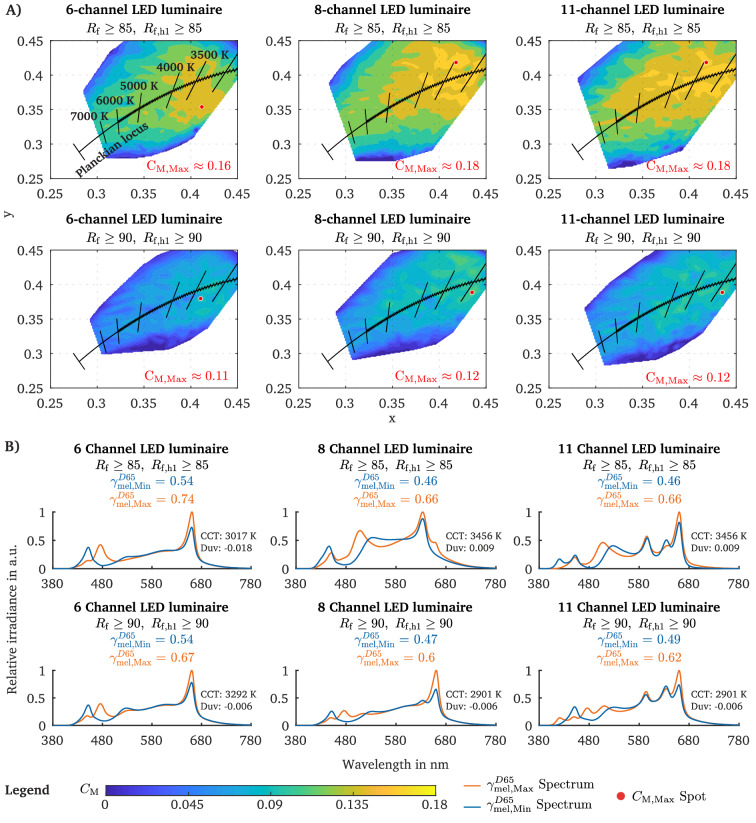
Melanopic metameric tuning limits (expressed in terms of melanopic Michelson contrast as derived from the maximum and minimum melanopic EDI, ${E}{}_{mel, Max}^{D65}$ and ${E}{}_{mel, Min}^{D65}$) at fixed photopic illuminance (250 lx). (**A**) The distribution of the melanopic Michelson contrast $C_{M}$ in the CIExy-2° colour space for three LED luminaire settings and two IES colour fidelity index R_f_ conditions by using the ${E}{}_{mel, Max}^{D65}$ and ${E}{}_{mel, Min}^{D65}$ value of each chromaticity coordinate at a fixed photopic illuminance of 250 lx. The red dots signal the CIExy coordinates at which the melanopic Michelson contrast is maximal across all CCT and Duv steps ($C_{M, Max}$). The value of $C_{M, Max}$ is reported in the lower left of each metameric map plot. (**B**) The maximum melanopic Michelson contrast $C_{M, Max}$ (red dot, panel **A**) is derived by using the ${E}{}_{mel, Max}^{D65}$ and ${E}{}_{mel, Min}^{D65}$ values of the spectral pair that corresponds to the red dot. The two spectra of the metameric pair, which maximise the melanopic Michelson contrast ($C_{M, Max}$), are plotted for each luminaire setting and colour fidelity conditions.

The ${\Delta {\gamma }}{}_{mel}^{D65}$ value is an intuitive way of visualising the melanopic tuning range for various metameric spectra. However, for evaluating the impact of a melanopic modulation and not only the ${\gamma}{}_{mel}^{D65}$ value at a particular CCT, it is important to also check the relative contrast of the melanopic EDI between the highest and lowest melanopic condition of a metameric pair. For this, we additionally mapped the melanopic Michelson contrast $C_{M}$ (see “Methods”) in the CIExy-2° colour space to visualise those chromaticity coordinate regions where spectra can be optimised that feature the highest relative change related to ${E}{}_{mel,Min}^{D65}$ (Fig. 3A, B).

The largest melanopic contrast can be considered either isolated for each CCT or globally across all CCT and Duv steps. The latter results in a $C_{M,Max}$ spot (see “Methods”), similar to the maximum in the previous analysis in (red point in Fig. 2A) for ${\Delta {\gamma }}{}_{mel}^{D65}$. The $C_{M,Max}$ peak contrast value reflects the melanopic modulation limit across all optimised chromaticity targets when using metameric spectra, indicating the technical possibility of a multi-channel LED luminaire configuration. Depending on the number of channels, the maximum melanopic Michelson contrast $C_{M,Max}$ across all CCT and Duv steps that can be reached, is between 0.16 and 0.18 when considering only those spectra that fulfil the third priority level (R_f_ $\ge$ 85, R_f,h1_ $\ge$ 85) of the colour fidelity (Fig. 3A). For example, with the 6-channel LED configuration (Fig. 3A), a $C_{M,Max}$ of 0.16 can be realised below the Planckian locus with a CCT of 3017 K (Duv − 0.018), allowing to modulate the melanopic EDI from approximately 135 lx to 185 lx (${\Delta {\gamma }}{}_{mel}^{D65}\approx 0.2$) without affecting the photopic illuminance (250 lx) and the chromaticity (Fig. 3B). With the 8-channel luminaire, a maximum melanopic Michelson contrast of 0.18 is possible at a CCT of 3456 K (Duv 0.009), with which the melanopic EDI can be modulated from approximately 115 lx to 165 lx, corresponding to a ${\Delta {\gamma }}{}_{mel}^{D65}$ of $\sim$ 0.2 (Fig. 3B). When using an 11-channel luminaire, the $C_{M, Max}$ peak is achieved at a CCT of 3456 K (Duv 0.009), resulting in the ability to modulate the melanopic EDI from 115 to 165 lx, corresponding to a ${\Delta {\gamma }}{}_{mel}^{D65}$ of ~ 0.2 (Fig. 3B). Thus, the level of the melanopic contrast $C_{M,Max}$ (red point in Fig. 3A) is not affected between the 8-channel and 11-channel luminaire at the priority level three of the colour fidelity (R_f_ $\ge$ 85, R_f,h1_ $\ge$ 85). Note that the reported maximum possible contrast across all CCTs or for an isolated CCT is given under our methodological condition, meaning that the metameric spectra must be within a distance of $\Delta u',\Delta v'\le 0.001$ from the chromaticity target. Slightly higher contrasts can be achieved if a larger tolerance to the chromaticity coordinate target is chosen, as this results in a larger number of spectra that can be considered for generating the metameric Michelson contrast. The applied tolerance of $\Delta u',\Delta v'\le 0.001$ was chosen so that the ellipses (Fig. 1B, step 2) around each chromaticity coordinate did not overlap, as we used a tight grid of optimisation targets along the Planckian locus (Fig. 1A).

The projection of the metamerism contrast values $C_{M}$ into the CIExy colour space (Fig. 3A) shows distinct areas in which a certain level of melanopic modulation can be achieved. For example, using the 6-channel luminaire, metameric spectra (R_f_ $\ge$ 85, R_f,h1_ $\ge$ 85) with a $C_{M}$ of at least 0.14 can be generated when choosing CCTs between 2700 to 4717 K within a Duv distance to Planck between − 0.027 to 0.015. However, by increasing the luminaire’s number of channels to eight, the chromaticity colour area in which a melanopic contrast of $C_{M}$ > 0.14 (R_f_ $\ge$ 85, R_f,h1_ $\ge$ 85) can be achieved widens (Fig. 3A). With the 11-channel LED luminaire, isolated islands of chromaticity regions between 2700 and 5102 K (Duv − 0.018 to 0.027) can be identified with which a melanopic Michelson contrast $C_{M}$ between 0.16 to 0.18 (R_f_ $\ge$ 85, R_f,h1_ $\ge$ 85) can be optimised. In the supplementary materials (Figs. S3, S4), we provide the maps of the non-visual metameric modulation limits without any colour fidelity condition, showing that the colour rendering is the major limiting factor when designing metameric spectra for non-visual purposes.

### Comparing the melanopic efficacy of metameric and non-metameric spectra

**Figure 4:**
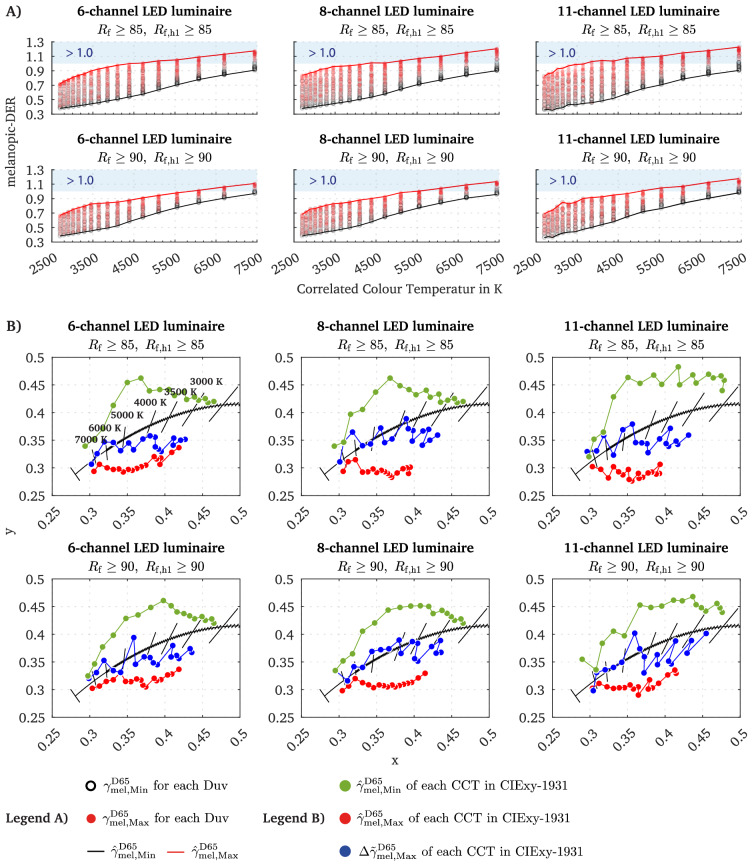
The melanopic DER as a function of CCT and the ideal chromaticity coordinates to reach spectra with the highest or lowest circadian efficacy. (**A**) Scatter plot of the ${\gamma }{}_{mel,Min}^{D65}$ and ${\gamma }{}_{mel,Max}^{D65}$ values as a function of the CCT. As for each CCT value several Duv distances to Planck were used as optimisation targets multiple solutions are available. The black lines indicate ${\widehat{\gamma}}{}_{mel,Min}^{D65}$, which is the lowest ${\gamma}{}_{mel,Min}^{D65}$ value for a particular CCT across all Duv steps. The red line refers to ${\widehat{\gamma}}{}_{mel,Max}^{D65}$, which is the largest ${\gamma}{}_{mel,Max}^{D65}$ value for each CCT across all Duv steps. (**B**) Identified ideal chromaticity coordinates for optimisation pipelines to get the spectra with the largest melanopic efficacy (${\widehat{\gamma}}{}_{mel,Max}^{D65}$, red line in (**A** and **B**), lowest melanopic efficacy (${\widehat{\gamma}}{}_{mel,Max}^{D65}$, black line in (**A**), green line in (**B**) or the largest melanopic tuning range (blue line in **B**) with ${\Delta \widetilde{\gamma }}{}_{mel}^{D65}$ through metamer spectra.

In this section, we analyse the determined melanopic DER ${\gamma }{}_{mel}^{D65}$ values for both, ${E}{}_{mel, Min}^{D65}$ and ${E}{}_{mel, Max}^{D65}$ as a function of CCT (Fig. 1D). For each CCT, multiple ${\gamma }{}_{mel,Min}^{D65}$ (black scatter) and ${\gamma }{}_{mel,Max}^{D65}$ (red scatter) values exist (Fig. 4A) since spectra were optimised for each CCT with about 32 different Duv distances (i.e., chromaticity targets per CCT, see Fig. 1 for explanation). With higher CCT, the distance between ${\gamma }{}_{mel,Min}^{D65}$ and ${\gamma }{}_{mel,Max}^{D65}$ distributions increases, and the respective scatter becomes narrower (Fig. 4A). Therefore, we can state, that above ~ 7000 K, the selection of the chromaticity coordinate (at steady CCT) does not significantly affect the melanopic efficacy of a spectral stimulus. At lower CCTs (< ~ 4500 K), highly circadian effective spectra could only be achieved with selected chromaticity coordinates.

This analysis (Fig. 4A) applies for non-metameric spectra, in which the Duv is a free parameter for each CCT. Such an aspect is crucial for comparing the melanopic tuning range of metameric spectra (steady chromaticity, Fig. 3) to non-metameric spectra with a fixed CCT, but variable Duv distance to Planck. From approximately 4600 K onwards (Fig. 4A), a spectrum can be generated that reaches a melanopic DER greater than one, regardless of the luminaire’s LED setting (R_f_ $\ge$ 85, R_f,h1_ $\ge$ 85). However, when applying a stricter colour fidelity index condition (R_f_ $\ge$ 90, R_f,h1_ $\ge$ 90), the CCT threshold with which a melanopic DER greater than one can be achieved is shifted towards higher CCTs to approximately ~ 6000 K (Fig. 4A). In lighting applications, it is often of interest to vary the melanopic efficacy at given CCT with no restriction on the choice of the Duv distance to the Planckian locus^54^, which would be not metameric but could result in the advantage of reaching higher melanopic efficacy. Therefore, we also plotted the trend line in Fig. 4A to denote the lowest melanopic DER value, ${\widehat{\gamma }}{}_{mel,Min}^{D65}$ and the largest value ${\widehat{\gamma }}{}_{mel,Max}^{D65}$ that can be generated for each CCT for non-metameric spectra. Note that the span width between the ${\widehat{\gamma }}{}_{mel,Min}^{D65}$ and ${\widehat{\gamma }}{}_{mel,Max}^{D65}$ lines in Fig. 4A should not be interpreted as metameric tuning range parameter with $\Delta {\gamma }{}_{mel}^{D65}$. For the latter only the highest and lowest melanopic DER for a particular Duv and CCT value needs to be considered (i.e. truly metameric spectra, see Fig. 1C, D).

From a practical point of view, the question arises which chromaticity coordinates between 2700 to 7443 K should be used to obtain spectra at the upper limit of ${\gamma }{}_{mel,Max}^{D65}$ (red line in Fig. 4A), as the melanopic efficacy of a spectrum could depend on its Duv distance to Planck. In Fig. 4B, the optimal chromaticity coordinates for spectral optimisation are highlighted (red points), making it possible to obtain the ${\widehat{\gamma }}{}_{mel,Max}^{D65}$ values (non-metameric). The ideal chromaticity coordinates for reaching ${\widehat{\gamma }}{}_{mel,Max}^{D65}$ are below the Planckian locus (6-channel: Duv range: -0.039 to -0.009). When applying the priority level two criterion for the colour fidelity index (R_f_ $\ge$ 90, R_f,h1_ $\ge$ 90), the ideal chromaticity targets for ${\widehat{\gamma }}{}_{mel,Max}^{D65}$ (6-channel) move closer to the Planckian locus within a Duv area between − 0.036 to − 0.006. As discussed in the first section, it must be assumed that the chromaticity regions that provide spectra with a maximum melanopic efficacy ${\widehat{\gamma }}{}_{mel,Max}^{D65}$ could differ from those that could achieve the largest melanopic tuning range $\Delta \widetilde{\gamma }{}_{mel,Max}^{D65}$ through metameric spectra (Fig. 1C). For example, in Fig. 4B, the chromaticity coordinates (blue points) are highlighted with which metameric spectra can be optimised to reach $\Delta \widetilde{\gamma }{}_{mel, Max}^{D65}$ for each CCT. These chromaticity points are closer to the Planckian locus within a Duv area between -0.027 and 0.009 (6-channel, R_f_ $\ge$ 85, R_f,h1_ $\ge$ 85). In contrast, the chromaticity location with which the lowest melanopic DER ${\widehat{\gamma }}{}_{mel,Min}^{D65}$ can be achieved (green points) are above Planck, regardless of the applied colour fidelity condition. Based on these results, however, we can state that the melanopic efficacy of the spectra depends significantly on the selected chromaticity coordinates and that spectra with a highly melanopic efficacy (non-metameric) are more likely to be found below the Planckian locus.

## Discussion

For a given chromaticity coordinate different spectra can be generated, each with unique visual and non-visual properties. Therefore, research recommendations for light related experiments advise researchers to specify the spectral power distribution in the reports^55–57^. The effect of metamerism has gained increased attention to modulate the melanopic stimulus strength and the circadian system without changing the chromaticity coordinate or illuminance. By optimising and analysing one of the largest spectral data sets, we showed for the first time that highly effective metameric spectra could be generated in specific chromaticity regions. Furthermore, we developed metamerism maps in the colour space for melanopic photoreception and circadian photoentrainment, useful for selecting chromaticity points for spectral optimisation pipelines to reduce the computational time. Additionally, we linked the chromaticity coordinates to the limits of metamer spectral optimisation, leading to a systematic evaluation of the CCT and non-visual response. Although higher CCTs could result in high melanopic EDIs^54^, melatonin attenuation can also be minimised by non-visual tuning using metamer spectra and hence minimising melanopic EDIs while maintaining the chromaticity.

The current study shows that for spectral stimuli with a CCT up to ~ 4500 K, a larger CCT generates a higher melanopic tuning range $\Delta {\gamma }{}_{mel}^{D65}$ when using the third priority level of the colour fidelity restriction (R_f_ $\ge$ 85, R_f,h1_ $\ge$ 85) when keeping the metameric spectra within a distance of $\Delta u',\Delta v'\le 0.001$ to the chromaticity target. However, for chromaticity coordinates corresponding to a CCT of ~ 4500 K and above, the effect slowly saturates and the melanopic stimulus can be modulated with a tuning range of at least 0.2 melanopic DER while keeping the photopic illuminance and chromaticity steady (R_f_ $\ge$ 85, R_f,h1_ $\ge$ 85) as shown in Fig. S2. When increasing the number of LED primaries in the luminaire (8-channel, 11-channel), higher $\Delta {\gamma }{}_{mel}^{D65}$ values can be achieved (Fig. S2, R_f_ $\ge$ 85, R_f,h1_ $\ge$ 85).

The studies by Allen et al. and Souman et al. indicated that a melanopic EDI difference $\Delta {E}{}_{mel}^{D65}$ of 52.9 lx or 122 lx can result in significant differences in melatonin-suppression^33,35^. Assuming that the metamers are within $\Delta u',\Delta v'\le 0.001$, we showed that, for a photopic illuminance of 250 lx, the melanopic EDI can be tuned by a maximum of $\Delta {E}{}_{mel, Max}^{D65}\approx$ 62.5 lx to 75 lx, corresponding to a melanopic DER tuning $\Delta {\gamma }{}_{mel,Max}^{D65}$ of approximately 0.25 to 0.30 (R_f_ $\ge$ 85, R_f,h1_ $\ge$ 85). However, it should be noted that our applied colour fidelity criterion is stricter than the previous conducted studies by Allen et al. and Souman et al., leading to a lower melanopic tuning range. The spectra as applied in the work of Souman et al. had a CRI below 80, which resulted in a high melanopic tuning range^35^. In principle, the defined colour fidelity criterion has a major influence on the reachable melanopic tuning level through metamers. For instance, if the colour fidelity condition is increased from (R_f_ $\ge$ 85, R_f,h1_ $\ge$ 85) to (R_f_ $\ge$ 90, R_f,h1_ $\ge$ 90), the largest $\Delta {\gamma }{}_{mel,Max}^{D65}$ across all CCTs are significantly decreased to 0.14, 0.17 and 0.19, depending on the luminaire configuration (Fig. S1). Increasing the colour fidelity can also induce other beneficial effects on humans, such as better visual comfort, better mood, higher daytime alertness and more deep sleep during the night^58^.

In order to generate a pair of two metameric spectra that feature a large tuning range $\Delta {\gamma }{}_{mel}^{D65}$, chromaticity points with a CCT towards ~ 7400 K should be preferred (R_f_ $\ge$ 85, R_f,h1_ $\ge$ 85), as $\Delta {\gamma }{}_{mel}^{D65}$ rises with higher CCT values (Fig. 2). As a rule of thumb, chromaticity coordinates below or slightly above (depending on the CCT) the Planckian locus should be preferred during spectral (metameric) optimisations where a large melanopic tuning range $\Delta {\gamma }{}_{mel}^{D65}$ is of interest (see Fig. 4B). Chromaticity coordinates above the Planckian should be chosen for lighting conditions in which the melanopic efficacy needs to be low (${\widehat{\gamma }}{}_{mel,Min}^{D65}$), i.e., during the last 3 h prior to bedtime in the evening (see Fig. 4B). Nevertheless, the ideal distance (Duv) to the Planckian locus depends mainly on the objective of the metameric spectral tuning: (i) optimising non-metameric spectra for a maximum melanopic efficacy (${\widehat{\gamma }}{}_{mel,Max}^{D65}$), (ii) optimising non-metameric spectra for a minimum melanopic efficacy (${\widehat{\gamma }}{}_{mel,Min}^{D65}$), (iii) maximising the metameric melanopic tuning range $\Delta {\gamma }{}_{mel}^{D65}$, (iv) or maximising the melanopic Michelson contrast. We have found that the chromaticity regions for achieving a maximum melanopic efficacy (non-metameric) for a given CCT are different from the regions in which the highest metameric tuning range (at fixed CCT and CIExy colour coordinates) can be generated, see Fig. 4B. As such, and pending the pursued objective, different chromaticity areas should be used in the spectral optimisation pipeline.

So far, we considered the light spectra’s colour rendition by using primarily the IES TM-30-20 colour fidelity index R_f_ metric. Additional visual metrics can also be applied together, like combining the colour fidelity with the vividness and colour preference, to further account for visual preference during metameric tuning. Such recommendations are provided in the IES TM-30-20 Annex E and could be applied in later works^40^. Note that the current work used the 2° colour matching functions (CMFs) to evaluate the chromaticity coordinates of the metameric spectra. Although the chromaticity coordinates of the metameric spectra are within the tolerance of $\Delta u',\Delta v'\le 0.001$ to the target chromaticity coordinate, still some colour differences might be visually perceived. Future work will need to verify the extent to which the melanopic contrasts and their distribution is affected when applying the 10°-CMFs. Therefore, in the sense of open science, we publish the optimised spectra (see data availability statement), as to enable custom analyses with extended metrics for other interested research groups. Furthermore, the analysis in this work assessed luminaire configurations that used two phosphor-converted white light LEDs in every setting. In few scientific applications, chromatic LEDs (or other) are applied without white LEDs^59–61^ to selectively trigger the photoreceptors^43,62^. For future work, it is recommended to verify whether the use of white-light LEDs has an advantage in the modulation of melanopic stimulus strength by means of metameric spectra. Compared to previous spectral optimisation publications, this work systematically relates metrics to each other based on a higher number of datasets rather than optimising individual spectra. The mapping of the melanopic metamer tuning limits is intended to serve as a recommendation basis in selecting distinct chromaticity regions for spectral optimisation procedures in integrative lighting research and industrial applications. These results are relevant for the promising field of metamerism in smart interior lighting^19^.

In the past, the science of spectral optimisation primarily focused on the trade-off between visual parameters of colour science and the luminous efficacy of radiation at given chromaticity coordinates to design a perfectly balanced light or to recommend an appropriate combination of chromatic LEDs for different purposes^32,63–69^. The introduction of globally standardised melanopic metrics^70^ or other ipRGC-related action spectra intended to evaluate the strength of light-induced circadian responses, which has led to additional objectives for spectral optimisation tasks. This circumstance has led to triangular optimisation constraints in which visual parameters were combined with non-visual metrics^46,71^ while maintaining a good luminous efficacy of radiation^30,39,72^. Today, integrative lighting is used as a synonym for illumination systems that target humans’ biological responses to light beyond vision, while considering the visual performance and visual preference^44^ in the construction of luminaires and spectral engineering. Here, we proposed and showed that metameric spectral design is a useful avenue to solve potential conflicts between an observer’s preference (or choice) of visual appearance of the light in terms of colour or chromaticity and a physiologically desired melanopic and circadian efficacy of the light. For instance, metameric spectra could be useful for night shift workers^73,74^, aiming to ensure an appropriate colour rendition and amount of light for visual tasks during the biological night while leaving the circadian system basically in biological darkness^75^ as to minimise circadian disruption. For evaluating the possibilities of metameric spectra to balance the visual preference of an observer with the non-visual aspects of light, future studies need to combine the criteria of preference, vividness, and fidelity from the IES TM30-20 Annex E and the impact of the age-dependent^76^ pre-receptoral filtering on such metrics, as in our work, we only considered the colour fidelity.

## Methods

### Multi-channel LED luminaire

The primary spectra of an existing 11-channel LED luminaire (Thouslite LEDCube) were measured using a Konica Minolta CS2000 spectroradiometer as a function of the duty-cycle and then interpolated. From the interpolated channel-spectra, a 6-channel, 8-channel and 11-channel LED luminaire was simulated in MATLAB MathWorks. The 6-channel LED luminaire had four narrowband LEDS with peak-wavelengths of 475 nm, 504 nm, 521 nm, 662 nm and two phosphor-converted white LEDs with a CCT of 4655 K and 2740 K. The 8-channel LED luminaire had narrowband LEDs with peak wavelengths of 450 nm, 465 nm, 504 nm, 521 nm, 638 nm, 662 nm and two phosphor-converted white LEDs (4655 K, 2740 K). The 11-channel LED luminaire had 8 narrowband LEDs with peak wavelengths of 419 nm, 450 nm, 457 nm, 504 nm, 521 nm, 597 nm, 638 nm, 662 nm, one phosphor-converted lime coloured LED and two white LEDs (4655 K, 2740 K).

The primary spectra of each luminaire configuration are reported in the appendix (Supplementary materials, Fig. S1). The selection of the LED combinations was made in such a way that with an increasing number of channels, more chromatic LEDs were added, which should result in a higher degree of freedom to maintain a chromaticity coordinate while modulating other melanopic metrics. During optimisation, the number of channels was specified as an adjustable parameter. This means that not necessarily all primary spectra are to be used. However, within a solution, the maximum number of used channels does not exceed the set limit. For example, it is possible that when using the 11-channel configuration, there could also be spectra/solutions that activate 9-channels rather than 11-channel.

### Spectral optimisation and metameric calculations

A uniform grid of 561 chromaticity coordinates (2700 K to 7443 K ± *Duv* 0 to 0.048) along the Planckian locus in the CIEu’v’-1976 colour space was used to define optimisation objectives (see Supplementary materials Fig. S1), similar to the authors’ previous publication^19,41^. For each chromaticity coordinate, spectra were optimised in a loop with six to eight repetitions, making sure to calculate enough metamers.

The spectra were optimised with the tolerance condition $\Delta u',\Delta v'\le 0.001$ for the chromaticity targets. Thus, spectra within $\Delta u'v'$ $\le$ 1.41 $\cdot 10^{-3}$ were defined as metamers, leading to a total of 490,068 optimised spectra. The spectra $X(\lambda )$ were initially optimised for an illuminance of 220 lx ± 2 lx. Then, all optimised spectra were linearly (re)scaled to reach a fixed photopic illuminance $E_{v}$ of 250 lx.

All optimisation results and scripts are available at the corresponding author’s GitHub repository. The spectra were optimised using a custom-developed heuristic optimisation procedure, which will be published separately. However, metameric spectra can be generated with any heuristic optimisation or by using other approaches as extensively discussed in detail by numerous publications and reviews^30–32,64,69,77–80^.

From the optimisation, a set of $N$ metamer spectra ${M}_{S} = [{X}_{1}(\lambda ), {X}_{2}(\lambda ),\dots ,{X}_{N}(\lambda )]$ for $X_{1}(\lambda ), X_{1}(\lambda ),\dots ,X_{N}(\lambda )\in \mathbb{R}^{401\times N\times C}$ with $C$ as the number of target chromaticity coordinates was derived. The non-visual impact of the optimised spectra $M_{S}$ was assessed by quantifying the melanopic stimulus strength in terms of melanopic EDI $\{{E}_{mel,i}^{D65}\}_{i= 1}^{C}$ for $E_{mel,i}^{D65}\epsilon {\mathbb{R}}^{N\times 1}$, using the formula (Eq. 1)

1 $$E_{mel}^{D65}=1/(1.3262/1000)\cdot \int_{380}^{780} s_{mel}(\lambda ) \cdot X(\lambda )\ d\lambda$$

with $s_{mel}(\lambda )$ as the melanopic action spectrum^36^ according to the CIE 026/E:2018. Next, for each chromaticity coordinate (*i*), the maximum melanopic tuning range was calculated across the metameric spectra with $\Delta E_{mel,i}^{D65}=|\mathrm{max }\ E_{mel,i}^{D65} - \mathrm{min }\ E_{mel,i}^{D65}|$ (see Fig. 1). As the melanopic EDI tuning range depends on the applied photopic illuminance, we additionally calculated the melanopic DER according to (Eq. 2)

2 $${\gamma }_{mel}^{D65} = {E}_{mel}^{D65}/ {E}_{v}$$

where $E_{v}$ is defined as our used photopic illuminance of 250 lx. The $\Delta {\gamma }{}_{mel,i}^{D65}$ values were interpolated and plotted using a heatmap in the CIExy-2° colour space (Fig. 2A). The spot with the highest possible melanopic tuning range across all chromaticity coordinates was calculated with $\Delta {\gamma }{}_{mel,Max}^{D65}= \mathrm{max}\ {\Delta \gamma }{}_{mel,i}^{D65}$. The respective $\Delta {\gamma }{}_{mel,Max}^{D65}$ values were marked as red dot in Fig. 2A. The metrics of the $\Delta {\gamma }{}_{mel,Max}^{D65}$ spectra are additionally stated in the supplementary materials for each luminaire configuration and colour fidelity condition. In Fig. 3A, we also plotted the melanopic Michelson contrast C_M,i_, which was calculated using the formula (Eq. 3)

3 $${C}_{M,i}=\frac{{E}_{mel,Max,i}^{D65}-{E}_{mel,Min,i}^{D65}}{{E}_{mel,Max,i}^{D65}+{E}_{mel,Min,i}^{D65}}$$

where ${E}{}_{mel,Max,i}^{D65}$ corresponds to $\mathrm{max }\ {E}{}_{mel,i}^{D65}$ and ${E}{}_{mel,Min,i}^{D65}$ to $\mathrm{min }\ {E}{}_{mel,i}^{D65}$ from the optimised metameric set $M_{S}$ of each chromaticity target. The procedure of calculating derived metrics is discussed in the first section of this manuscript (see Fig. 1). For calculating the IES TM-30-20 colour fidelity metrics from the spectra, we used the LuxPy library^81^, developed by Kevin Smet. All optimized spectra, the calculated metrics, the MathWorks MATLAB and Python scripts are available online at the main author’s GitHub repository (see data availability statement).

## Supplementary Information

Supplementary Information.

## Data Availability

The data that support the findings of this study are available at the following GitHub repository: https://github.com/BZandi/Metameric-Spectra.
