# Supplementary material for: Optimising metameric spectra for integrative lighting to modulate the circadian system without affecting visual appearance
Source: Sci Rep. 2021 Nov 30;11:23188. doi: 10.1038/s41598-021-02136-y (PMC8633386; doi:10.1038/s41598-021-02136-y)
Supplement: Supplementary file 1 — Supplementary Information. [file 41598_2021_2136_MOESM1_ESM.pdf]

Supplementary Materials

Optimising metameric spectra for integrative lighting to modulate the circadian system without affecting visual appearance

Babak Zandi[1),*], Oliver Stefani[2)], Alexander Herzog[1)], Luc Schlangen[3)], Quang Vinh Trinh[1)], Tran Quoc Khanh[1)]
[1)]Technical University of Darmstadt, Department of Electrical Engineering and Information Technology, Laboratory of Lighting Technology.
[2)]University of Basel, Transfaculty Research Platform Molecular and Cognitive Neurosciences (MCN), Centre for Chronobiology.
[3)]Eindhoven University of Technology, Intelligent Lighting Institute, Eindhoven, Netherlands.
[*]Corresponding author: zandi@lichttechnik.tu-darmstadt.de

Additional materials are available at the project's GitHub repository:
https://github.com/BZandi/Metameric-Spectra

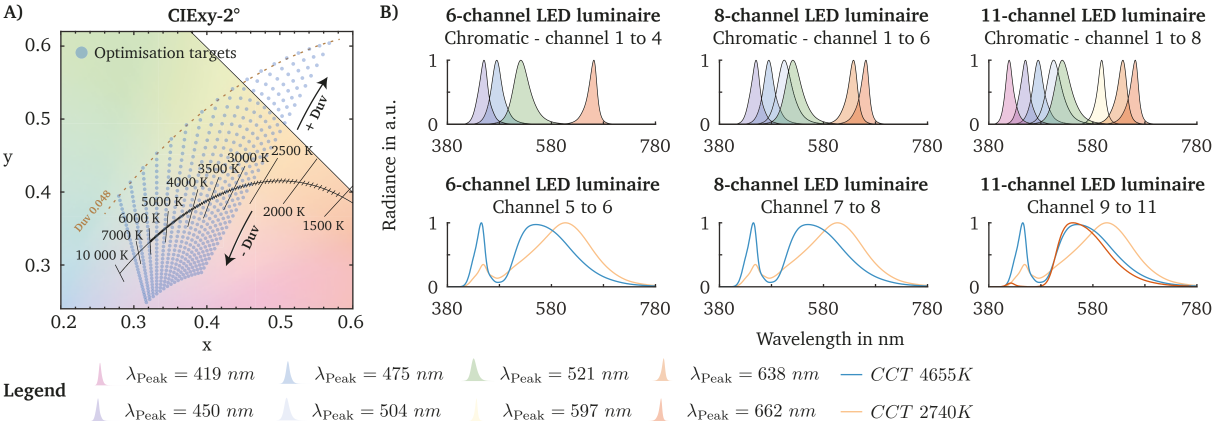

Figure S1 Optimisation targets in the CIExy-2° colour space and the primary spectra of the multi-channel LED setups. Figure was reprinted from Zandi, B., Eissfeldt, A., Herzog, A., and Khanh, T. Q. (2021). Melanopic Limits of Metamer Spectral Optimisation in Multi-Channel Smart Lighting Systems. Energies 14, 527. doi:10.3390/en14030527. License CC BY 4.0.

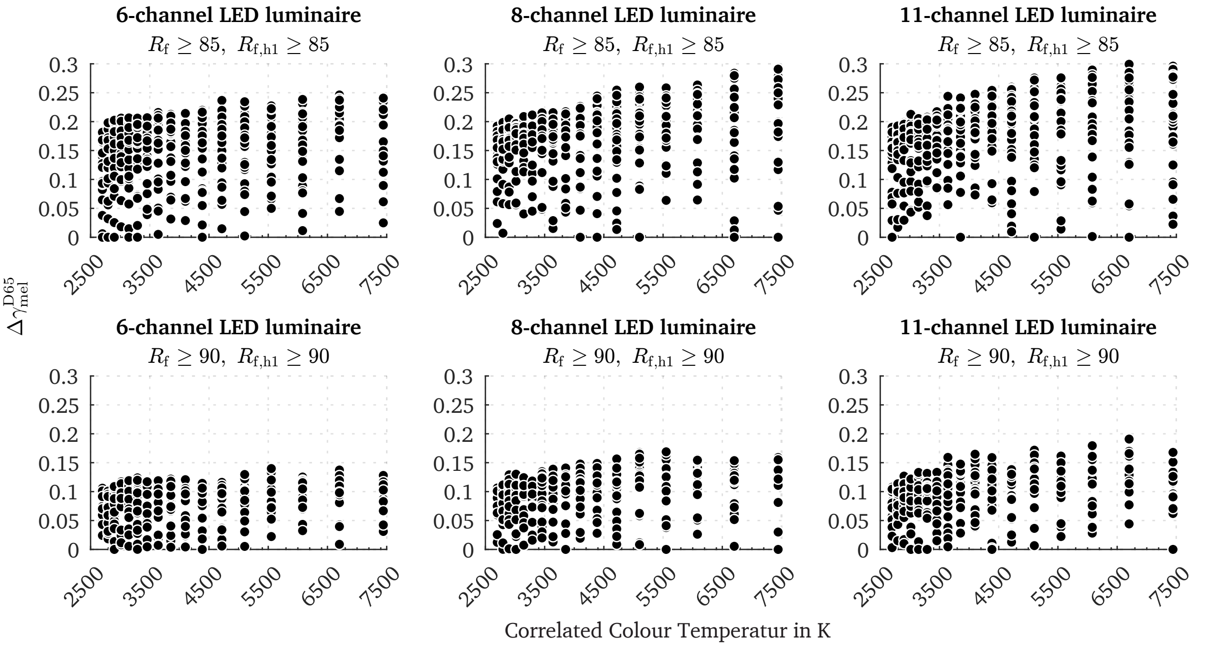

Figure S2 Calculated melanopic tuning range $\Delta\gamma_{mel}^{D65}$ for each chromaticity points plotted against the CCT calculated following the procedure as outlined in Figure 1 of the main manuscript.

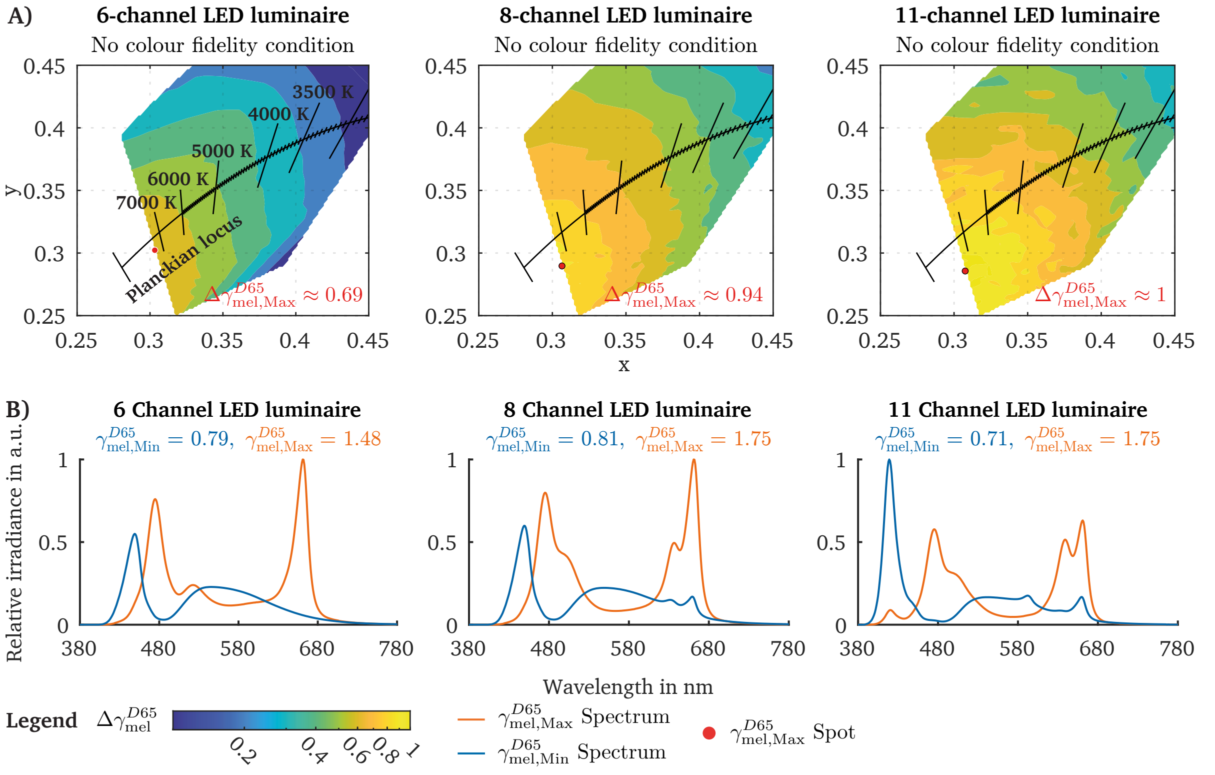

Figure S3 Melanopic tuning range with metamer spectra at fixed photopic illuminance (250 lx) without a colour fidelty condition. **A:** the melanopic DER tuning range in the CIExy-2° colour space for three LED luminaire settings and no colour fidelity conditions. The red dots signal the CIExy coordinates at which the melanopic tuning range is maximal ($\Delta\gamma^{D65}_{mel,Max}$). **B:** The respective relative metamer spectra with the maximum and minimum melanopic DER for the $\Delta\gamma^{D65}_{mel,Max}$ spots across all chromaticity targets, which are highlighted in the CIExy colour space in panel **A**.

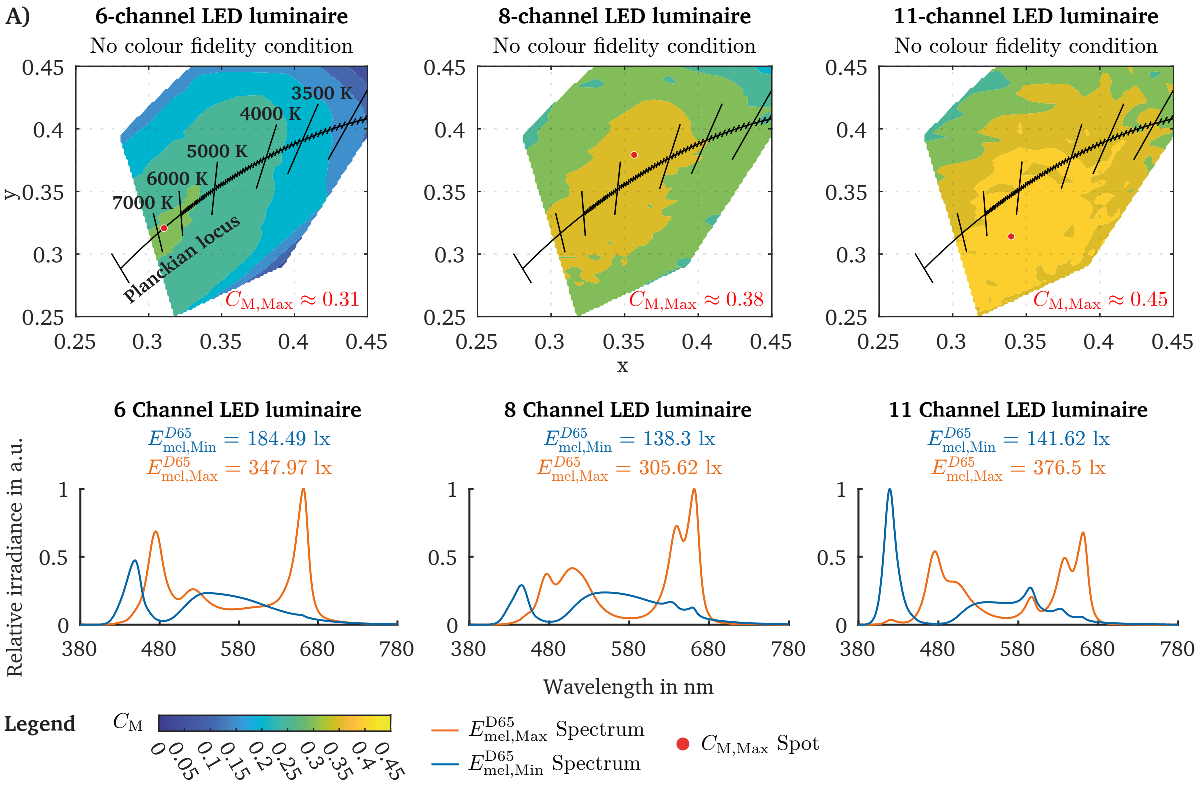

Figure S4 Melanopic tuning limits with metamer spectra and fixed photopic illuminance (250 lx) when using the melanopic Michelson contrast without a colour fidelity condition. **A:** Mapping the melanopic Michelson contrast C_M in the CIExy-2° colour space for three LED luminaire settings by using $E^{D65}_{mel,Max}$ and $E^{D65}_{mel,Min}$ value of each chromaticity coordinate. The red dots signal the CIExy coordinates at which the melanopic Michelson contrast is maximal ($C_{M,Max}$). **B:** The respective relative metamer spectra with the maximum and minimum melanopic EDI for the $C_{M,Max}$ spots across all chromaticity targets, which are highlighted in the colour space.
